# Weight variation increases the risk of death during the intensive phase of treatment among MDR-TB patients: A retrospective study

**DOI:** 10.12669/pjms.39.4.7025

**Published:** 2023

**Authors:** Abdul Majeed Akhtar, Shamsa Kanwal, Sufia Majeed, Wasif Majeed

**Affiliations:** 1 Dr. Abdul Majeed Akhtar, Ph.D. University Institute of Public Health, The University of Lahore, Lahore, Pakistan; 2 Dr. Shamsa Kanwal, Ph.D University Institute of Public Health, The University of Lahore, Lahore, Pakistan; 3 Dr. Sufia Majeed, MBBS. Department of Medicine, Mayo Hospital, Lahore, Pakistan; 4 Wasif Majeed, M.Phil. Institute of Applied Psychology, The University of Punjab, Lahore, Pakistan; 5 Institute of Applied Psychology, The University of Punjab, Lahore, Pakistan

**Keywords:** Intensive phase, Multidrug Resistance Tuberculosis, Mortality, Weight

## Abstract

**Objective:**

To estimate the predictors of death during intensive phase of Multidrug resistant tuberculosis treatment according to the weight of patients at the time of diagnosed.

**Methods:**

A retrospective study was conducted at three public hospitals in the Lahore, Punjab region, namely Jinnah Hospital, Mayo Hospital and Gulab Devi Hospital on 1,496 patients receiving treatment for MDR-TB from January 2018 to December 2020. Data were collected from electronically nominating and recording system of the hospitals. Data were fitted to Cox proportional hazards regression model with 95% confidence interval (CI) to evaluate the associations between predictors of death and weight of MDR-TB patients during the intensive phase of treatment.

**Results:**

This analysis revealed a MDR-TB mortality rate of 30% and the mortality rate due to MDR-TB during the intensive phase of treatment was 23%. The variables related to increased mortality among underweight patients were age more than 60 years (HR: 0.398, 95% CI: 0.314-0.504) , diabetes (HR: 1.496, 95% CI: 1.165-1.921), current smoking (HR: 0.465, 95% CI: 0.222-0.973), history of MDR-TB (HR: 0.701, 95% CI: 0.512-0.959) and culture positive at the time of diagnosed (HR: 0.499, 95% CI: 0.379-0.659) during the intensive phase of treatment.

**Conclusion:**

The high mortality rate among the underweight MDR-TB patients during the intensive phase of the treatment requires the nutritional support for malnourishment and ensured a close follow-up of the elderly patients with co-morbidities as well as family history of Tuberculosis.

## INTRODUCTION

Tuberculosis is a major public health issue caused by Mycobacterium tuberculosis which increases a larger number of deaths from infectious diseases worldwide. WHO Reported in 2018, approximately 10.0 million population diagnosed from TB globally and the prevalence was 132 cases per 100,000 people.^1^ The origin of approximately 61% TB cases in the Eastern Mediterranean WHO region is Pakistan which making it one of the leading contributors to the globally TB burden.^2^

Multidrug resistant tuberculosis (MDR-TB) is known as tuberculosis (TB) with resistance to two first line anti-TB drugs i.e., isoniazid and rifampin which is usually associated with more expensive treatment, longer hospitalization, and higher mortality.^3^ Some studies have reported that body weight variation had significant impact on the TB treatment outcome especially in drug-sensitive TB.^4^,^5^ Pakistan is ranked fourth with high burden of MDR-TB globally.^5^ Pakistan has more than 30 programmatic management of DR-TB (PMDT) which are providing free of cost diagnostic and treatment facilities to all DR-TB patients.^6^ National Tuberculosis Control Program, Pakistan reported mortalities among TB patients is 34 per 100,000 population per year. However, the high death and lost to follow up rates are generally cited as the major problems in achieving better cure rates.^7^,^8^

National Tuberculosis Program, Pakistan (2019) defined cured as treatment completed without evidence of failure and two consecutive cultures taken at least 30 days apart are negative in the continuation phase whereas died are those patients who dies for any reason during the course of treatment. The total treatment duration for MDR-TB patients is 18 to 20 months which may be adjusted according to the patient response to treatment therapy. The treatment regimens for MDR-TB patients have an injectable drug during the first six months of treatment after the culture conversion is known as intensive phase.^7^ This study aimed to estimate the predictors of death during intensive phase of MDR-TB treatment according to the weight of the patients at the time of diagnosed.

## METHODS

A retrospective study was conducted at three public hospitals in the Lahore, Punjab region, namely Jinnah Hospital, Mayo Hospital and Gulab Devi Hospital. Records of Electronic Nominal Record System (ENRS), laboratory reports and medical charts of all registered MDR-TB patients from January, 2018 to December, 2020 were reviewed after the approval of this study from Provincial Bioethics Committee Punjab dated 20/12/2017, Ref: 2017/3441. The patients with confirmed diagnosis of MDR-TB by Drug Sensitivity Test (DST) were included in the study while the patients having incomplete information, lost to follow up, other causes of death, and transferred out from Lahore were excluded from the study. A total of 1496 patients from 2455 were selected for the study by applying non-probability convenience sampling technique. Patients were followed until cured or died due to MDR-TB during study period. Once the positive sputum test for rifampicin and isoniazid resistance were identified by the microbiology laboratory, patients were registered at hospital. Patients’ demographic characteristics, comorbid conditions and other possible risk factors for mortality of MDR-TB were recorded.

Date of death was the response variable and explanatory variables were demographic characteristics (age, gender and residence), behavioural predictor is smoking, clinical characteristics (baseline weight, body mass index, co-morbidities, radiological findings, time of sputum culture conversion, history of TB treatments, previous TB treatment outcome and number of previous MDR-TB treatment).

The demographic characteristics are reported according to body mass index (kg/m^2^) category (Normal weight (18.5-24.9), underweight (<18.5) and overweight (≥ 25)). The Pearson chi-square test was used for categorical data. Treatment outcome according to weight was expressed as the mean± standard deviation. A Cox proportional hazards regression model with 95% CI was applied to evaluate the associations between factors of death and weight of MDR-TB patients during the intensive phase of treatment. A p-value of < 5% was set to be statistically significant. All data management and analyses were performed using the SPSS 26 software.

## RESULTS

**Fig.1:**
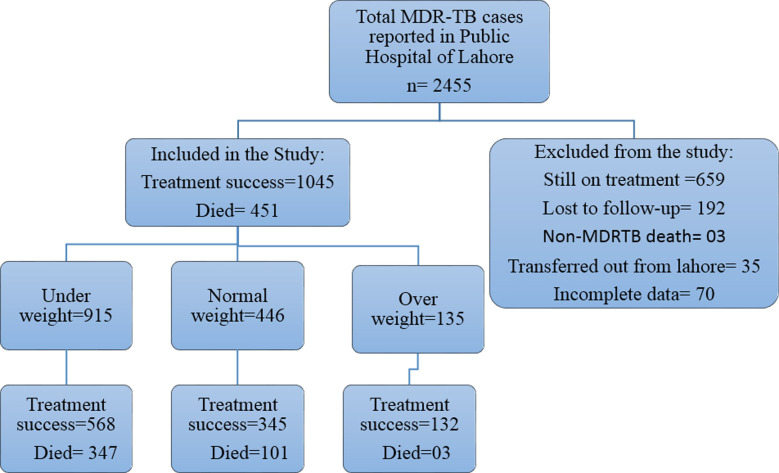
Study Population, Multidrug resistance tuberculosis patients.

From January 2018 through December 2020, we found the record of 1496 patients with microbiologically confirmed multidrug resistance tuberculosis (MDR-TB), 451 died due to MDR-TB during the study and 1045 cured after completion of their treatment of MDR-TB. Of the study patients, 915 (61%) were under weight and 38% cured while 23% died due to MDR-TB in the category of underweight, 446 (30%) had normal weight (23% cured and 07% died) and 135 (09%) were overweight (8.8% cured while 0.2% died) (Fig.1).

The demographic and clinical characteristics of patients according to BMI are summarized in Table-I. Of these, 813 (54%) were younger than 30 years, 50% were males, 88.4% were current smokers followed by diabetes (27%) and liver diseases (9%), history of tuberculosis (7%), history of MDR-TB (14%). The majority (98%) were from smear positive most of whom (84%) were culture positive at the time of diagnosis. Younger than 30 years and older than 60 years (p = 0.000), diabetes (p = 0.000), history of tuberculosis (p = 0.007), culture positive at the time of diagnosis (p = 0.045) showed high significance among the underweight MDR-TB patients.

**Table-I:** Characteristics of Multidrug Resistance tuberculosis patients according to BMI at enrollment for MDR-TB.

BMI (kg/m^2^)					

Characteristics	N (%)	Underweight (n= 865)	Normal (n= 446)	Overweight (n=185)	p
***Age (year)***					0.000^*^
<30	813(54)	555	186	72	
30-59	616(42)	275	244	97	
≥60	067(04)	35	16	16	
***Gender***					0.000^*^
Male	754 (50.4)	352	274	128	
Female	742 (49.5)	513	172	57	
***Smoking status***	1322 (88.4)				0.255
Current smoker	47(3.14)	760	403	159	
Former smoker	127(8.84)	30	8	9	
Never smoker		75	35	17	
***Diabetes mellitus***					0.000^*^
No	1092(73)	685	304	103	
Yes	404(27)	180	142	82	
***Liver disease***					0.138
No	1359(91)	794	395	170	
Yes	137(09)	71	51	15	
***History of Tuberculosis***					0.007^*^
No	1397(93)	799	430	168	
Yes	97(07)	66	16	17	
***History of DR-TB***					0.000^*^
No	1291(86)	769	377	145	
Yes	205(14)	96	69	40	
***Extra-pulmonary TB***					0.915
No	754(50)	440	222	92	
Yes	742(50)	425	224	93	
***Acid-fast bacilli- Smear***					0.031^*^
Positivity					
No	29(02)	12	9	8	
Yes	1467(98)	853	437	177	
***Culture Test Positivity***					0.045^*^
No	229(15)	149	54	26	
Yes	1267(85)	716	392	159	
***Timing of death***					0.000
During interim phase	334(22)	257	77	3	
After interim phase	117(08)	90	24	0	

The mean values of the weight at the time of diagnosis of the MDR-TB patients according to the outcome variable in the treatment is shown in Table-II. The mean weight significantly dropped within the first eight months of the treatments and the rate of mortality increased (P=0.000) among the underweight patients. In the overweight patients a trend was seen favoring treatment after culture conversion during the treatment. The total mortality rate of the study patients was 30% (451/1496) while the mortality rate among MDR-TB patients before the intensive phase was 23% (337/1496).

**Table-II:** Summary statistics of treatment outcome according to weight (kg) with respect to BMI of MDR-TB patients in Lahore, Pakistan

Treatment outcome according to weight (Kg)	BMI		

	Underweight n(Mean ± SD)	Normal weight n(Mean ± SD)	Overweight n(Mean ± SD)
Alive	568 (39 ± 7)	345 (51 ± 8)	132 (61 ± 8)
Death before interim phase	257 (38 ± 8)	77 (47 ± 10)	03 (57 ± 7)
Death after interim phase	90 (43 ± 9)	24 (49 ± 8)	0

Total	915 (40 ± 9)	446 (50 ± 8)	135 (59 ± 10)

A Cox proportional hazards model was used to identify risk factors for death during the intensive phase in MDR-TB patients (Table-III). The risk factors associated with death during the intensive phase included aged ≥ 60 years (HR: 0.398; 95% CI, 0.314-0.504), current smoker (HR: 0.465; 95% CI, 0.222-0.973), Diabetes mellitus (HR: 1.496; 95% CI, 1.165-1.921), history of DR-TB (HR, 0.701; 95% CI, 0.512-0.959), AFB smear positivity (HR, 0.313; 95% CI, 0.179–0.546) and culture positivity (HR, 0.499; 95% CI, 0.379–0.659). Factors associated with mortality after culture conversion included age ≥ 60 years (HR, 0.364; 95% CI, 0.270-0.491), former smoker (HR, 0.469; 95% CI, 0.255-0.863), liver disease (HR, 0.663; 95% CI, 0.499-0.882) and history of TB (HR, 1.713; 95% CI, 1.101-2.666).

**Table-III:** Predictors of Mortality among MDR-TB patients before and after intensive phase at public hospitals of Lahore.

Variables	Died during the Intensive phase		Died after Intensive phase	

	AHR	95% CI	AHR	95% CI
***Age (year)***				
< 30	1		1	
30-59	1.103	0.764-1.592	0.717	0.460-1.117
≥ 60	0.398	0.314-0.504*	0.364	0.270-0.491*
***Gender***				
Male	1		1	
Female	0.915	0.722-1.159	1.127	0.859-1.479
***Smoking status***				
Never smoker	1		1	
Current smoker	0.465	0.222-0.973*	0.673	0.330-1.374
Former smoker	0.642	0.360-1.146	0.469	0.255-0.863*
***Diabetes mellitus***				
No	1		1	
Yes	1.496	1.165-1.921*	0.797	0.512-1.240
***Liver disease***				
No	1		1	
Yes	1.307	0.899-1.899	0.663	0.499-0.882*
***History of Tuberculosis***				
No	1		1	
Yes	0.847	0.503-1.426	1.713	1.101-2.666*
***History of MDR-TB***				
No	1		1	
Yes	0.701	0.512-0.959*	0.642	0.457-0.903*
***Extra-pulmonary TB***				
No	1		1	
Yes	1.161	0.916-1.472	0.819	0.623-1.077
***Acid-fast bacilli- Smear Positivity***				
No	1		1	
Yes	0.313	0.179-0.546*	0.344	0.182-0.650*
***Culture Positivity***				
No	1		1	
Yes	0.499	0.379-0.659*	1.559	1.088-2.234*

* Significant at p<0.05.

## DISCUSSION

In this study, we analyzed risk factors for mortality in MDR-TB patients registered for the treatment in three public hospitals of Lahore, Pakistan. The overall mortality was 30% and the mortality rate during the intensive phase was 23%; this frequency was similar to results from previous studies conducted in Pakistan and Colombia while lower from the India.^6^,^9^,^10^ When death due to MDR-TB was considered, underweight was associated with an increased risk of mortality during MDR-TB treatment in our study. This association between underweight and the active tuberculosis has been thoroughly mentioned in scientific literature.^11^-^13^

Previous history of MDR-TB and the family history of TB has been similarly associated with a higher risk of death in our study. Patients with multiple regimens of anti-tuberculosis treatment might increase the antibiotic resistance with the development of MDR-TB.^14^ Some studies have found a significant association between history of MDR-TB and higher risk of mortality.^15^,^16^

Diabetes and age less than 30 years as well as more than 60 years develop the factors of mortality as it decreases cellular immunity, which favors the progression of the disease.^17^ This association has been demonstrated in the literature.^18^,^19^ Diabetes as a comorbidity among MDR-TB patients showed the high frequency.^20^ Akhtar et al reported that smoking was significantly associated with a higher risk of MDR-TB in the Lahore, Pakistan.^21^ The present study indicated that smoking was not significantly associated with the risk of mortality, but current and heavy smoker was significantly associated with a higher MDR-TB mortality during the intensive phase of treatment. Furthermore, the main impact of underweight and current smoking on mortality occurred within the first eight months of MDR-TB treatment. The findings in this study showed that underweight and former smoking was an independent risk for MDR-TB mortality after the intensive phase of MDR-TB treatment. Similar results can be viewed from the published articles.^22^-^24^

### Limitations:

It was retrospective study so some information of patients like drugs used for comorbid conditions, ototoxicity, complementary therapies and adverse events were not observed.

## CONCLUSION

As the results identified that the association between weight of the MDR-TB patients and mortality which are public health problems that contribute to improving treatment of MDR-TB. The high mortality rate among the underweight MDR-TB patients during the intensive phase of the treatment requires the nutritional support for malnourishment and ensured a close follow-up of the elderly patients with co-morbidities as well as family history of Tuberculosis.

### Authors’ Contribution:

**AMA** did review and final approval of manuscript.

**SK** designed and did statistical analysis & editing of manuscript, is responsible for integrity of research.

**SM** did data collection and manuscript writing.

**WM** entered the data and drafting.
